# Through each other's eyes: initial results and protocol for the co-design of an observational measure of adolescent-parent interaction using first-person perspective

**DOI:** 10.3389/frcha.2023.1214890

**Published:** 2024-03-04

**Authors:** Nicky Wright, Rebecca M. Pearson, Danielle Crook, Alice Bond, Tom Jewell

**Affiliations:** ^1^ Department of Psychology, Manchester Metropolitan University, Manchester, United Kingdom; ^2^ Centre for Academic Mental Health, Population Health Sciences, Bristol Medical School, University of Bristol, Bristol, United Kingdom; ^3^ Department of Primary Care & Mental Health, University of Liverpool, Liverpool, United Kingdom; ^4^ Research Department, Cheshire and Wirral Partnership NHS Foundation Trust, Chester, United Kingdom; ^5^ Florence Nightingale Faculty of Nursing, Midwifery & Palliative Care, King’s College London, London, United Kingdom; ^6^ Great Ormond Street Hospital NHS Foundation Trust, London, United Kingdom

**Keywords:** parenting, measurement, family interaction, parent-adolescent, observation, adolecence, adolescent-parent

## Abstract

**Background:**

Current observational methods to understand adolescent-parent interaction are limited in terms of ecological and content validity. We outline initial results and a protocol for future work from a programme of work to: (1) establish a new method for data capture of adolescent-parent interaction at home using wearable cameras and; (2) develop a new relevant and comprehensive observational micro-coding scheme. In Part 1, we report our completed preliminary work, comprised of an initial scoping review, and public engagement work. In Part 2, we present a protocol for the development of the new measure.

**Methods:**

Part 1—We searched Pubmed for existing observational measures of adolescent-parent interaction for the scoping review. We also undertook public engagement work utilising a mobile research van, taken to multiple locations around Bristol, UK to engage with a variety of populations through interactive methods. Part 2—Our protocol describes plans for: (1) A systematic review of the psychometric properties of observational measures of adolescent-parent interaction; (2) Focussed public engagement workshops; (3) Harmonisation of information from existing coding schemes and literature with information from public engagement with adolescents and parents; (4) A pilot study to assess the acceptability and feasibility of the method; (5) Development of a coding scheme in consultation with expert and lay panels, and through real-life application to recorded videos from a pilot sample.

**Results:**

Scoping review: we identified 21 adolescent-parent observational schemes, of which eight used micro-coding and 13 used globalcoding schemes. The majority of micro-coding schemes were not developed specifically for adolescents. Most studies used conflict or problem-solving tasks, which may not adequately capture positive adolescent-parent interactions. The mobile van event received views from 234 young people and/or parents. Families were positive about taking part in research using headcams. “Trust” and “understanding” were most frequently reported as important adolescent-parent relationship constructs.

**Conclusions:**

This work represents the first attempt to truly co-design a method to assess parenting in adolescence. We hope to develop an observational measure using novel technological methods that can be used across a range of research and therapeutic settings.

## Introduction

Adolescence constitutes a crucial developmental period in which most mental health disorders typically arise (1). It also represents a period of relational transition with growing sense of identity and autonomy or independence from parents (2–4), which is accompanied by increases in adolescent-parent conflict (5) and decreases in closeness (6). Mental health disorders are rising in young people at an alarming rate (7) and child and adolescent mental health services are unable to meet the level of demand for treatment (8). Family interventions are recommended in the treatment of adolescent mental health disorders including conduct disorder, eating disorders and depression (9–11). However, understanding of adolescent-parent interaction is limited, particularly regarding micro processes underlying relationships (12). Such processes are key to relational and behavioural interventions and may be best understood from observing and analysing behavioural interactions.

There is a large body of high-quality observational evidence from studies showing that specific parenting constructs in early childhood promote later cognitive and socio-emotional development, and these findings have been successfully translated into behavioural interventions which improve relationships (13). However, such understanding is lacking in adolescence. In addition, in child, adolescent and adult romantic relationship interaction research there is a greater focus on negative and problematic interaction patterns (14, 15). Thus, there is a need for observational measures of adolescent-parent interaction that can assess relational strengths as well as difficulties, with well-established ecological and content validity. The measures are needed to allow researchers to capture real life interactions and then to make meaningful observations from them to aid better understanding of relationships during adolescence. Secondly once established to provide tools to assess and evaluate relational change in clinical settings as well as to provide feedback to families using the tools directly in interventions. It is key to achieve this so that tools are accessible to families, researchers and professionals in educational, psychology or psychiatric settings. Such measures could have several applications: firstly, to inform the development of relational interventions for clinical populations with mental health problems; secondly, to understand family processes in the general population; and thirdly, to better understand family relationships and how that links to mental health and other outcomes through use in large international prospective cohort studies. The present paper aims to set out a program of research to develop such a measure and is structured in two parts. In Part 1, we report preliminary work we have already conducted, comprised of an initial scoping review and public engagement exercise. In Part 2, we report a protocol for the next steps we will undertake to develop the measure. However, to begin with, we present an overview of the field, highlighting some of the key methodological limitations of prior research.

### Overview of observational measures of adolescent-parent interaction

Firstly, we review what observational work has been conducted with parents and adolescents to date. By “observational” we mean methods where behaviour between parents and adolescents is observed and recorded to allow objective behavioural analysis. One aspect of observational analysis concerns how the interaction is ***elicited and captured*** (i.e., *where* and how it is recorded and *what* the family is doing) and the second aspect concerns how the behaviour and relationship is then ***assessed or*** “***coded***” from the observation. We explore both improvements to methods of data capture and the coding of interactions here.

We begin by scoping existing measures. Researchers have utilised observational measures of adolescent-parent interaction since at least the 1980s (16), yet to our knowledge there has never been a systematic review focused on such measures. Twenty years ago, Aspland and Gardner (17) provided a helpful narrative review of observer-rated measures of parent-child interactions, highlighting issues including differences in task (structured vs. unstructured), setting (laboratory vs. home) as well as discussing issues of validity and reliability. However, this review did not aim to identify all existing schemes, and only a few measures were discussed for purposes of illustration. Locke and Prinz (18) reviewed all measures they identified as assessing discipline and nurturance developed in the preceding two decades. Only three observational measures were identified for the adolescent period, and six identified for early adolescence. The authors highlight the need for validation studies in different cultural groups, as well as the importance of research into the ethnicity of coders as a variable which may influence coding. Jewell et al. (19) conducted a systematic review of attachment measures in middle childhood and adolescence, but only identified one scheme assessing observed adolescent-parent interaction, the goal-corrected partnership adolescent coding system (GPACS) (20), with all other observer-rated schemes assessing the child on their own, typically using attachment interviews.

Marshall et al. (21) recently conducted a systematic review of measures of adolescent-parent conflict processes and their measurement, identifying 568 measures from 467 articles, of which 54 measures utilised observer ratings. The approach to inclusion was generous, with many included studies providing scant details of the observer-rated task or measure [Marshall (2023) personal communication]. Moreover, the aim of this review was to analyse the ways in which adolescent-parent conflict has been conceptualised and measured (e.g., frequency of disagreement, duration of conflict), and the psychometric properties of measures were not assessed. Finally, there have been two systematic reviews conducted on the psychometric properties of observer-rated parent-child interaction measures for children: Cañas, Ibabe & De Paúl (22) assessed measures for 0–12-year-olds at risk of child abuse and neglect, whilst Gridley et al. (23) investigated measures for 0–5-olds used in randomized controlled trials. Both reviews highlighted a lack of strong validity evidence for such measures.

In summary, it is known that observational-measures of adolescent-parent interaction have been utilised frequently in research, yet the literature has not been reviewed, and thus the full scope of the field, as well as the psychometric properties of measures, are currently unknown. Furthermore, the extent to which previous observed measures have conducted in depth micro-analysis (where all behaviours and sequences between behaviours are explored) remains unclear because few previous studies include micro-coding systems across all behavioural domains.More in depth micro-coding is important given the potential for such micro codes to guide the development of more specific and person-centred interventions.

### Limitations of existing observational measures

Whilst there are advantages to observational measures, there are challenges too, with perhaps the most widely acknowledged difficulty being the length of time needed to capture and then code recorded or live interactions. Less commonly discussed are several important threats to the validity of such measures both in terms of data capture and coding of content: firstly, a lack of ecological validity due to lab-based observation; secondly, insufficient content validity evidence due to non-involvement of parents and adolescents in the development of measures; and thirdly, a lack of evidence of content validity and measurement invariance across different cultures and changing times.

### Ecological validity and methods of observation (data capture)

#### Home or Lab settings

In terms of ecological validity, existing observational measures of adolescent-parent interaction typically involve visits to a laboratory to take part in a structured task, such as a conflict discussion [e.g., Allen et al. (24)]. Evidence suggests that behaviour under “laboratory conditions” does not reflect behaviour at home (25, 26).

Lab assessments and researcher-administered home assessments may lead to reactivity effects, whereby people change their behaviour due to the presence of the researcher or knowledge that they are being recorded. There is limited evidence examining this: Gardner (25) reviewed the literature based on audio-recorded assessments, which suggested no evidence of reactivity effects, but this did not compare researcher-present to researcher-absent conditions. When assessments from lab and home observations are compared, lower levels of negative behaviours are shown in the lab (15) which may reflect reactivity effects in the lab setting.

**Figure 1:**
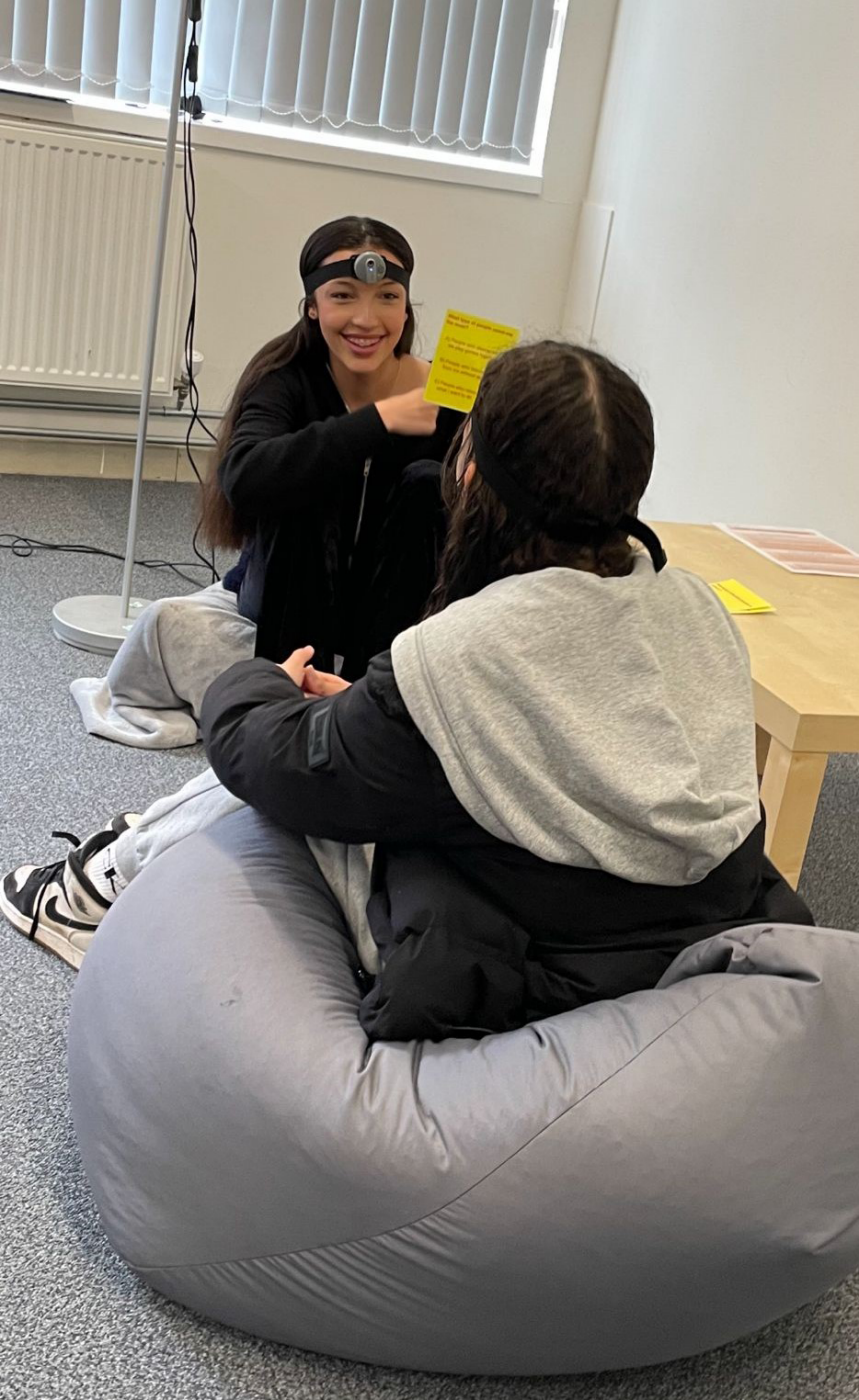
An example of the two teens wearing head-cameras completing an interaction task.

In addition, even though home settings improve ecological validity as compared to lab settings, in most situations a researcher is still present and has visited at a scheduled time. We have previously provided evidence for improved ecological validity of observational assessment conducted using wearable cameras with families of infants. Lee et al. (26) found that when families with infants self-recorded interactions with wearable cameras alone, as compared to the same dyads interacting at home but recorded by a researcher, more “negative” and socially undesirable maternal behaviours were observed. Footage recorded from wearable cameras also has a major advantage in that it captures a “first-person” perspective on an interaction, in contrast to the “third-person” perspective recorded from a static camera positioned to film an interaction from a side-view. Firstly, the first-person perspective allows for superior capture of faces and eye gaze; secondly, there is the potential for the development of therapeutic uses for such footage, such as video feedback techniques in which a therapist reviews footage with a parent or adolescent, allowing them to see themselves “through the eyes” of the other person. Figure 1 provides a photograph of the wearable cameras.

#### Tasks to elicit interactions

Another key to ecological validity is not only where the interaction is recorded but *how* the interaction is evoked. Very early work observing parent-child interaction was conducted at home involving multiple visits from researchers who observed and coded naturally occurring family behaviour “live” (27). The time-consuming nature of this led to the use of “structured” tasks designed to elicit behaviours of interest. Dishion and Stormshak (14) conceptualise these tasks as assessing either relationship quality or behavioural management. Relationship quality tasks in infancy and childhood are free-play or separation and reunion tasks. From childhood to adolescence, planning discussion tasks (such as planning a fun family activity) are typically used to assess relationship quality. In childhood, “clean-up” tasks are used to assess behavioural management and in later childhood and adolescence problem solving or conflict discussion tasks are used. The vast majority of adolescent studies use problem solving or conflict discussion, and these tasks have been shown to elicit relevant negative interaction behaviours when they use real rather than hypothetical scenarios (14). There is conflicting evidence on whether these behaviours elicited in the lab generalise to behaviours observed in unstructured settings at home. Behaviours observed in home settings are more strongly linked to ratings of psychopathology than behaviours observed in a lab, with the strongest associations found for unstructured tasks in the home (25). Behavioural coding across structured and unstructured tasks within the same dyads also show only modest agreement.

Although the traditional view of adolescence as a time of high conflict with parents is not supported by evidence, there is an increase in more minor conflicts in early and middle adolescence (5, 6), meaning that conflict discussions are a relevant context for assessing adolescent-parent interaction. These discussions allow observation of negative conflict processes such as criticism, hostility and coercion, and also more positive behaviours such as validation, support, and listening which may reflect adaptive conflict resolution strategies (28). However, conflict discussions are less likely to evoke other positive behaviours such as affection or shared enjoyment (14, 15). In addition, given that engaging in a conflict discussion generates negative emotion and increased physiological arousal (29–31) this context may not be best suited to assess positive adolescent-parent interaction quality. Tasks which may evoke more positive interaction qualities are needed. Such tasks may also be more acceptable and ethical in the context of parents and adolescents completing and recording tasks at home without the presence of a researcher.

### Threats to the validity of existing measures that code or assess observations

Content validity refers to the extent that the *content* of an instrument adequately reflects the construct to be measured (32). For adolescent-parent interaction measures, the perspectives of parents and adolescents on content validity are strikingly absent in the development of measures. Instead, measures appear to have typically been developed in a “top-down” fashion by researchers based on theoretical constructs such as attachment [e.g., Obsuth et al. (20)] or coercive family cycles (33, 34). The lack of involvement of parents and adolescents hampers not only the content validity of measures, but also represents a missed opportunity to gain valuable suggestions on the acceptability and feasibility of tasks. Involving parents and adolescents through co-design of a measure through participatory methods would help to strengthen the content validity of a measure, in terms of both the task and the scope of the construct/s to be measured. Such work can be combined in iterative fashion during the *substantive phase* of validation (35) and feed into other key early tasks such as mapping concepts and reviewing literature.

Moreover, insufficient attention has been paid to the role of culture in developing measures, both across and within countries. For instance, content validity cannot be assumed for minoritized groups within a culture, such as ethnic minorities. Adolescent-parent relationships will be strongly influenced by cultural context, thereby complicating attempts to define norms for concepts such as communication. This points to the need for careful validation work, which can include consultation with stakeholders, qualitative interviews, as well as formal studies of measurement invariance, when seeking to validate a measure for a specific population. In addition, the culture of researchers may influence their coding: for instance, Yasui and Dishion (36) found inter-rater reliability to vary as a function of the ethnicity of coders, as well as of the concordance between the ethnicity of the coders and the families being rated.

Finally, we must consider the neglected concept of time in relation to content validity. Many measures of adolescent-parent interaction were developed several decades ago. Notwithstanding the absence of parent/adolescent involvement in their development, we can ask: do such measures retain their validity across time, or does validity evidence require continual renewal? Before answering this question, it is worth going back to first principles and considering the meaning of validity, which refers to the degree to which evidence and theory support the interpretations of test scores for proposed uses of tests (37). Thus, validity is *not a property of a measure*; rather, statements about validity should be made with regard to specific uses of a measure (37). Researchers are likely to be familiar with the notions of cross-cultural validity and measurement invariance—put simply, the need to evaluate the performance of a measure in a new context, such as a translated measure used in a new country. Returning to the question of validity across time, we suggest that adolescent-parent relationships have changed so much in Western societies that they represent a *new cultural context* when compared to families of several decades ago. Consequently, whilst the validity evidence for all measures has the potential to degrade over time, we suggest that measures aiming to assess interactions between family members are particularly vulnerable to this due to the significant changes that have impacted adolescent-parent interaction in developed countries. Such changes include: a trend towards older age for completing transitions out of adolescence such as family formation or ending education (38); the arrival of new technology and social media, giving rise to “digital parenting” (39); and broader societal changes in parent-child relationships such as increased communication and quality-time, reduced authoritarian control and corporal punishment, and decreased parental self-efficacy (40). In summary, construct validation is an ongoing process (41) which requires evidence to justify specific uses (37); viewed in this light, existing observational measures of adolescent-parent interaction are far-from convincing in terms of content and construct validity.

### Micro and global approaches to coding of observational content

**Figure 2:**
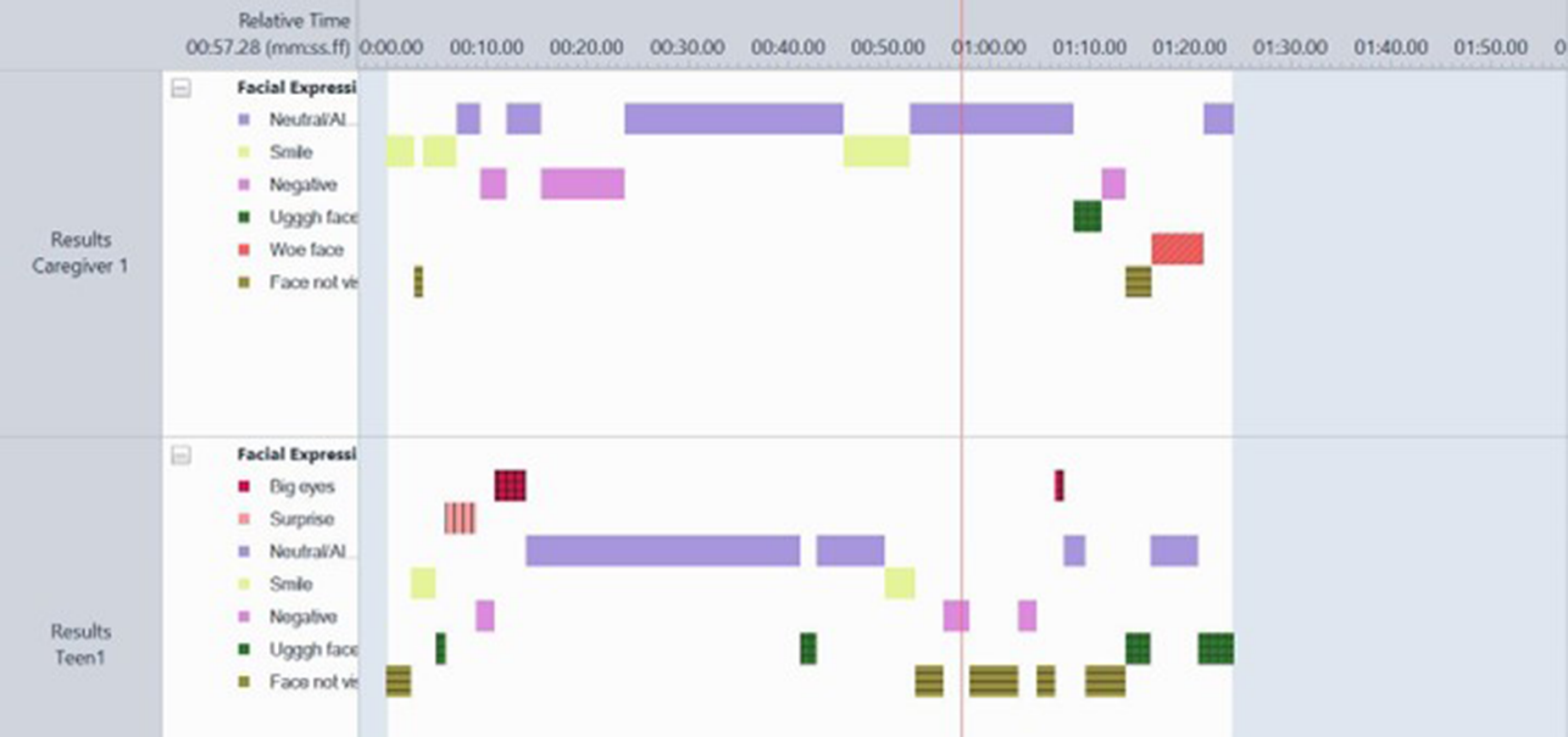
Example of micro coded data on the behavioural domain of facial expressions. The number and duration of each behaviour is visualised by the colour blocks in Observer XT.

Most observational coding schemes of parent-child interaction assess “global” constructs. Global ratings require the observer to form overall impressions from multiple different units of behaviour and consider frequency, intensity and duration within an entire interaction to make a numerical rating on a single scale. For example, in the global rating of maternal sensitivity, the most well-known construct in infancy, a researcher makes a numerical rating based on their global impression of how well the mother identifies, interprets and appropriately responds to her child's cues (42). This requires specific and time-consuming training to be able to assess reliably with baselines which were often developed several decades ago. Micro-coding, in contrast, focuses on individual units of behaviour and allows examination of moment-to-moment temporal sequences of behaviour (43, 44). This approach considers more subtle interactional processes and allows the identification of specific behaviours which may precede desirable or less desirable “target behaviours”, thus providing unique (and flexible) information which may be readily translated into intervention to promote positive parent-child interactions (44). Micro-coding allows tracking of frequency, sequence and duration of interactive behaviour (see Figure 2). Some micro-coding approaches focus on individual units of behaviour such as facial expression [e.g., the Facial Action Coding System; FACS; (45)]. Individual units of behaviour permit automated coding, which considerably reduce the time and costs associated with observational data. Most micro-coding approaches outside of infancy reflect behavioural groupings which integrate across multiple different behaviours (e.g., facial expression, bodily movements, speech) and require consideration of the context. In the SPAFF (Specific Affect Coding System), Coan and Gottman (46) refer to these codes as “gestalt behaviour codes” and conceptualise them as unobservable latent variables which are directly observed via specific behavioural indicators. For example, “validation” is observed with the indicators of agreement/apology, summarising, and head nodding with eye contact.

Pearson and colleagues (47) developed a comprehensive micro-coding scheme for parent-infant interaction including both discrete and gestalt behaviours from a review of all existing coding schemes and have shown that reliability can be easily achieved (47, 48). The scheme is applied using the Observer XT [version 16 (49)] software which permits coding of observational data on a timeline. Figure 2 shows an example of micro coded data on the behavioural domain of facial expressions. The number and duration of each behaviour is visualised by the colour blocks using Observer XT software.

#### Limitations of existing micro-coding and considerations for adolescence

Micro-coding also has limitations and particular considerations regarding its suitability for adolescent behaviour. Most notably, in micro-coding in infancy the context of each specific behaviour is not usually captured. For example, consider a negative or sad face by a mother. The context of this facial expression could lead to very different meanings and thus targets for interactions. For example, it may reflect a negative emotional state in the mother and lack of warmth or may reflect emotional mirroring by the mother (which is likely to be highly sensitive). This can be lost in micro-coding even if combinations or sequences are used.

In infancy micro-coding schemes, one facial expression category that is highly predictive is a “woe” face. As first described by Beatrice Beebe (50), this is an exaggerated sad face that is specifically linked to compassion/sympathy. It does not necessarily mirror a sad face in the child but instead marks an understanding by the caregiver which signals sympathy rather than negativity. Given adolescents’ vastly more sophisticated social and cognitive development relative to infants, the ability of micro-codes to capture contextual subtleties such as humour or sarcasm may be a particular challenge for coding schemes of adolescent-parent interaction.

### Summary and aims

There is a need for methods to collect observational data which are acceptable, feasible and ecologically valid, and coding schemes which adequately characterise modern adolescent-parent relationships, with strong content and construct validity. Such methods can advance our understanding of parenting in adolescence and have the potential to inform the development of new interventions, based on observed interactions. This could include therapies that directly utilise observational material within the course of treatment, such as video feedback, or the adaptation of existing approaches, such as parenting groups or family therapy, utilising evidence gleaned through observational research. New methods are required due to the limitations of available measures, including substantial threats to content and construct validity.

The programme of work described here aims to develop a new observational measure of adolescent-parent interaction by: (1) conducting a scoping review of the literature to identify existing adolescent-parent observational coding schemes and extracting all interaction codes; (2) conducting public engagement work with relevant stakeholders (adolescents, parents and professionals) to identify currently relevant constructs; (3) harmonising the “top-down” information from the existing literature with the “bottom-up” information from relevant stakeholders to generate a micro-coding scheme for adolescent-parent interactions and suitable scenarios or tasks to record behaviour; (4) carrying out a feasibility study of the wearable camera methodology and initial validation of the coding-scheme in a pilot study of adolescents and parents. We believe this is the first attempt to generate a tool to measure adolescent-parent interaction which combines both the traditional “top down” approaches taken in research and clinical practice with “bottom up” information from adolescents and parents.

### Overall project aims

1) Identify existing concepts important in adolescent-parent interactions from both Public Patient Involvement (PPI) and existing literature.
2) Develop an observational system to capture adolescent-parent interaction behaviour based on the existing literature and co-creation methods.
3) Explore feasibility and acceptability of wearable cams with adolescents and their parents at home.
4) Develop a protocol (including tasks) which (a) can be conducted at home (b) allows capture of relevant footage and (c) evokes / captures relevant and authentic domains of the relationship.

Initial completed work presented in this paper:

1) Scoping review of existing adolescent-parent observational measures (links to aim 1 and 2)
2) Scoping public engagement with adolescents and parents of methods and concepts (links to aim 2)

Next steps to be described as protocols in this paper:

1) Systematic review of measurement properties of existing coding schemes (links to aims 1 and 2).
2) Construct definition through focused public engagement workshops (links to aims 1, 2 and 4).
3) Harmonisation of information from existing coding schemes with information from public engagement with adolescents and parents (links to aims 1, 2 and 4).
4) Assess acceptability and feasibility of data capture by wearable camera technology (links to aims 3 and 4).
5) Development of a coding scheme in consultation with an expert panel and lay/PPI panel and through real-life application to recorded videos from a pilot sample (links to all aims).

Table 1 links the aims and objectives of the project to each piece of work outlined below, and summarises the output and current status of each.

**Table 1:** Aims, objectives and linked activities for the project.

Aim	Goal	Activity	Output	Status	Next step	
Identify known constructs and measures in parent-teen relationships	Scope existing concepts and schemes	Scoping review	21 schemes identified, very few micro- or recent.	Complete	Map to concepts identified below	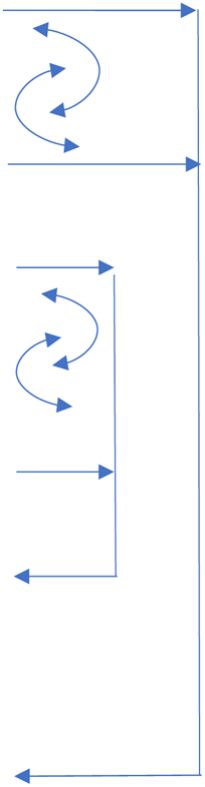
Identify new and relevant constructs in parent-teen relationships	Hear from communities what concepts are important	Mobile public engagement	Identification of most important/frequent: Trust and Understanding.	Complete	Harmonise with constructs in literature above	
Identify task/situation to elicit record behaviour	Scope what previous studies have done	Scoping review	Found most studies were in “lab” or home with a researcher present and conflict tasks.	Complete	Guide adaptions based on current needs	
Identify task/situation to elicit and record behaviour	Hear from communities what situations/tasks could work and whether wearable cameras are acceptable.	Mobile event	Protocol developed.	Complete	Integrate with existing work and design protocol	
Feasibility and acceptability of protocol	Design study procedures to test feasibility and acceptability of protocol	Full standard operating procedure and protocol developed and launched in cohort study	Protocol launched ( https://www.youtube.com/watch?v = LLxWI8dplbg&t = 1s )	In progress	Data collection and analysis	
Develop micro-coding scheme	Map concepts such as trust and understanding to more specific observed behaviours	Specific targeted workshops	Specific operationally defined behavioural signatures of important constructs.	Planned		

The arrows represent the links between the different activities, with double headed circular arrows representing activities which influence each other.

## Part 1—Completed ground work

### Methods

#### Scoping review to identify existing coding schemes, relevant behaviours, and indices of relationships

To identify papers with observed adolescent-parent interaction data we conducted a search in Pubmed using the terms “parent adolescent observed interaction coding behaviour” (and all related synonyms, see Supplementary file S1 for full search terms). Abstracts were screened to identify papers which included observation of parent-child interaction with samples with a mean child age of between 10 and 19 years. The methods sections of relevant papers were then checked to identify coding schemes to be included. Coding schemes were not included if they focussed on a very specific scenario (e.g., discussions about cigarette smoking; parent-child interaction within a therapy session) or included codes only on whole-family interaction.

#### Public engagement/co-creation

Scoping public engagement: we conducted a PPI mobile research van event in multiple locations around Bristol, UK, in August 2022 (51) with the aim of engaging with a variety of populations including those typically considered “hard to reach”. Researchers spoke to young people and their parents to find out their views on what they considered to be the characteristics of a “good” adolescent-parent relationship and key concepts were recorded using whiteboards. Parents and young people were invited to try out the wearable cameras and give their views on taking part in research using them. They were also asked what tasks or everyday scenarios would be acceptable and would best capture their relationship interactions at home. Initial conversations were used to create a voting system where subsequent families voted on their chosen task.

### Results

#### Scoping review of coding schemes

The search terms identified 128 papers. Of those, 44 papers included adolescent-parent interaction observational coding, reflecting 9 micro- and 13 global- coding schemes. An additional 4 micro- and 2 global- schemes were identified from the reference lists of the Marshall et al. (21), Locke & Prinze (18) and Jewell et al. (19) reviews and from reviewing reference lists of relevant papers. One global scheme was added from a preliminary search ran for the systematic review of measures (described below). Given our focus on micro-coding and space constraints, we will provide a more detailed account of micro-coding schemes in this paper. These initial summaries for this paper focus on the characteristics of the schemes rather than a description or synthesis of the specific concepts assessed in the codes.

##### Summary of micro-coding schemes

Table 2 displays the identified micro-coding schemes. Columns summarise characteristics including: whether they were developed for adolescents; their history; what tasks they were developed for; and the type of codes contained. Micro-coding can be “event” or “timing” based. In event-based coding, once a behaviour is coded, for example when a parent smiles, they will be coded as smiling until that changes; that is, codes are updated according to new “events”. In time-based coding, the behaviour is assessed within specific time windows. For example, in a time-based coding scheme using two second intervals, at two seconds a parent could be coded as smiling; at four seconds, if the parent is still smiling, then they will again be coded as such. We use the term “behaviour groups” to describe codes that involve combinations of individual units of behaviour. For example, “fear” in the Specific Affect Coding System [SPAFF (46)] is a combination of a range of different verbal characteristics (e.g., shifts in frequency in speech, speech disturbances), body movements (e.g., gestures or fidgeting) and facial movements (e.g., gulping and biting lip).

**Table 2:** Summary of the characteristics of the identified adolescent-parent micro-coding schemes.

Author	Name of scheme	Summary of codes	Made for adoles­cents?	History/theoretical background	Developed for specific population?	Tasks developed for
Dishion et al. (52)	Family process code (FPC)	Behaviour groups represented by a 2 × 2 grid, one axis content (grouped by verbal, vocal, nonverbal, physical contact, and compliance behaviour; 9 positive, 9 negative, 7 neutral) and the other valence (Exuberant, Positive, Neutral, Negative, Unrestrained Negative, and Sad Affect). 25 codes. Event-based^a^.	No—Developed for families	Developed by the Oregon Social Learning Centre group on the Family Interaction Coding System (FICS) by Reid (27). This was developed using observations of clinical families and social learning theory principles, particularly Patterson's (1982) coercive cycle (34). FICS is used with children only. This was revised into the Interaction Coding System (53) to use event-based, instead of timed interval coding, and into the Multidimensional Observations of Social Adjustment in Children (MOSAIC) (54). This was done after studying “functioning” families to better sample prosocial behaviour and behaviours shown in problem solving behaviour. MOSAIC codes were split into content, valence, activity and context. This was then reduced into the Family Process Code by reducing the behaviours and the FPC has been used with adolescents.	No	A range of tasks or unstructured settings
Dishion et al. (55)	Relationship process code (RPC)	Behavioural groups represented by a 2 × 2 grid, one axis content (grouped by verbal, nonverbal, physical) and the other valence (positive, neutral, negative). 13 codes. Event-based or time-sampling (15 s).	No—Developed for families and peer relationships	Developed from the FPC (see FPC for history) which was revised into the Interpersonal Process Code [Rusby et al. (56)] by eliminating less frequently occurring behaviours and behaviours that are difficult to code, as they require high levels of inference.	No	A range of tasks or unstructured settings
Dumas (57)	INTERACT	Five categories of codes (actor, behaviour, setting, adverb, and valence) combined according to specific syntactical rules to form discrete observation strings. Timing based.	No—Developed for families	Based on social learning theory principles of coercion and reciprocity, used to explain reinforcement of aggressive and depressive behaviour.	No	Unstructured and problem-solving tasks
Gottman & Krokoff (58), revised by Coan & Gottman (46)	The specific affect coding system (SPAFF)	Behaviour groups: Affect (based on verbal content, voice tone, context, facial expressions, gestures and body movement combined; 5 positive, 12 negative, 1 neutral). 18 codes. Timing-based (1 s with 3 s window).	No—Developed for families	Developed based on the Couples Interaction Scoring System [CISS; Gottman, 1979 (59)] and the Facial Action Coding System (FACS) (45) to include “gestalt” behavioural groups, which integrates multiple individual units of behaviour, including verbal content. Developed for adult romantic relationships and then applied to families.	No	Problem solving tasks
Hauser et al. (16)	Constraining & enabling coding system (CECS)	Verbal behavioural codes: two cognitive constructs (cognitive constrainers and cognitive enablers), two affective constructs (affective constrainers and affective enablers), and an interpersonal process construct (discourse change). Event based.	Yes	Developed based on theory on adolescent ego development, specifically the psychoanalytic work of Helm Stierlin (60), who was concerned with the ways in which family members inhibit adolescents who are attempting to individuate from the family.	No	“Revealed differences” paradigm (parent and adolescent complete a moral judgement test separately then their different responses are revealed and discussed)
Hops et al. (44)	Living in familial environments (LIFE)	10 behavioural codes for affective content (e.g., happy and angry) and 27 codes for verbal content (e.g., validation and affection). Event based.	No—Developed for families	Social learning theory principles, with a focus on differentiating aggressive and depressive behaviour. Reviewed literature to identify behaviours which distinguished depressed and non-depressed individuals. Also included aggressive and prosocial behaviours. Used codes from the Marital Interaction Coding System [MICS; Hops et al. (61)], the MOSAIC, and the FICS.	Families of depressed mothers.	Problem-solving tasks
Jabson et al. (62)	Simple affect coding system (SACS)	Behaviour groups: Affect (tone, facial affect, and body posture and/or orientation.no verbal content). 5 domains (Positive affect (7), validation (4), anger/disgust (12), distress (13) and neutral (2). 38 total codes. Event-based^a^.	No—Developed for families	Developed by the Oregon Social Learning Center (OSLC) group based on the work of Ekman et al. (FACS) and Gottman et al. (e.g., SPAFF) on emotional display.	No	Not specified
Notarius et al. (63) adapted by Aiken et al. (64), for adolescents	Codebook for marital and family interaction (COMFI)	Behaviour groups. 6 basic categories (Problem-solving facilitation (4), problem-solving inhibition (7), emotional validation (6), emotional invalidation (9), self-disclosure (2) and depressive statements (2)). 30 total codes. Event based. Everything said in the interaction is transcribed and utterances are divided into thought units and rated.	No—Developed for families and couples.	Developed by the marriage and family studies group. Integrates features of the Couples Interaction Scoring System (CISS); Affective Style [Doane et al. (65)] the MICS which is based on social learning theory principles (61); Weiss & Summers (66) and KPI [Hahlweg et al. (67)]. Developed so that affect, content and function are integrated into single codes	No	Problem-solving tasks
Peterson et al. (68)	Relationship affect coding systems (adolescent) coding manual (RACS)	Behaviour groups: 4 categories. Verbal behaviour, which includes 3 sub-categories: conversation (3), behaviour change (3) and vocal (1). Physical behaviour (3). Affect behaviour (5). “Off codes” (4). 19 total codes. Event based.	Yes	Built by combining the RPC (see RPC section for history) and the SACS (see SACS for history). Verbal codes from RPC reduced, affect codes expanded based on SACS.	No	Problem-solving tasks and all other discussion tasks included the Family Assessment Task; FAST Dishion & Kavanagh (69): including: planning an activity, parental encouragement of growth (e.g., academic growth), positive recognition of family members.
Robin & Foster (70)	Parent-adolescent interaction coding system—revised (PAICS-R)	Six verbal behavioural groupings: Commands/Put Downs, Defends/Complains, Problem Solution, Facilitates, Defines/Evaluates, and Talks. Event/Utterance based (but utterance defined as ongoing until the other person speaks)	Yes	Developed from the MICS, which is based on social learning theory principles and family systems theory. Specifically draws on behaviours which define the skills, communication style, and interaction patterns believed to determine resolution of parent-adolescent conflict (Robin, 1980) (71).	No	Problem solving tasks
Rodriguez et al. (72)	Contingency coding system (CCS)	Verbal behavioural groups based on utterances (8): reflection, reframe, expansion, disclosure, solicit, provision of information, imperative, and validation, plus other, un-codable and no code. Each utterance received a topic maintenance code (i.e., whether the mother “maintained” or “changed” the topic of conversation). Utterances did not receive multiple category codes (i.e., utterances could not be coded both as a reflection and solicit). Event/utterance based.	No—Developed for children and adolescents	Developed based on research regarding parents’ conversational discourse and management during their children's early language development, such as the use of repetitions, recasts, and expansions of children's speech (e.g., Fey et al. (73); Lasky & Klopp (74); Saxton (75)).	Child/ adolescent cancer patients	Conversations about cancer
Stubbs et al. (76)	The family and peer process code (FPPC)	Behaviour groups represented by a 2 × 2 grid, one axis content (grouped by verbal, vocal, nonverbal, physical contact, and compliance behaviour; 8 positive, 9 negative, 7 neutral) and the other valence (Happy, Caring, Neutral, Distressed, Aversive and Sad). 24 codes plus a withdrawal qualifier. Event-based^a^.	No—Developed for families and peer relationships	The FPPC is a combination of the FPC, RPC and the Peer Process Code [PPC; Dishion et al. (77)] which was based on the FPC and includes additional codes relevant to peer interactions.	No	A range of tasks or unstructured settings

^a^ Event based means once a behaviour is coded, for example when a parent smiles they will be coded as smiling until that changes i.e., codes are updated according to new “events”. In time-based approaches the behaviour is assessed within specific time windows, so at 2 s coded as smile, at 4 s if the parent is still smiling, they will again be coded as smiling.

Scheme history: Most of the identified schemes were either directly developed within or were influenced by work in the Oregon Social Learning Centre (OSLC), using social learning theory (SLT) principles, particularly Patterson's cycle of coercion (34). As described in the table, the initial Family Interaction Coding System [FICS (27)], which has not been used with adolescents so not included in the review, has been developed and extended into several other schemes which were identified (Family Process Code (FPC), RACS, Peer Process Code (PPC) (52, 68, 72)). This work is largely based on clinical samples of children and families with behavioural problems, but also some studies with general population samples with further developments of the scales. Schemes identified in this review were also influenced by schemes developed using social learning theory principles to assess conflict resolution in adult marital relationships such as the Marital Interaction Coding System [MICS (61)] and the Couples Interaction Scoring System [CISS (59)]. The Living in Familial Environments (LIFE), Codebook for Marital and Family Interaction (COMFI), Parent-Adolescent Interaction Coding System-Revised (PAICS-R) (44, 63, 70) all identified as being developed from these marital relationship schemes. The PAICS-R also draws on family systems theory. The LIFE scheme included consideration of the SLT processes involved in maintaining depression (reciprocity). Another scheme developed for children, INTERACT (57), draws on both coercion and reciprocity models from social learning theory. Another school of schemes which focus more narrowly on affect were developed from Ekman's work on coding facial expression [SPAFF & Simple Affect Coding System (SACS)] (58, 62). One identified scheme specifically focused on conversational discourse in relation to language learning (Contingency Coding System; CCS) (72). Finally, one scheme drew on psychoanalytic theory on adolescent ego development (Constraining & Enabling Coding System; CECS) (16).

Code types: Most schemes were designed to use event-based coding; the INTERACT and SPAFF are timing based; and the Relationship Process Code [RPC (55)] was described as suitable for both event and timing-based coding. All the identified schemes described “behaviour group” codes, and none focussed on coding individual units of behaviour (such as just facial expression or visual attention). Schemes from the OSLC tend to separate verbal from non-verbal in their behavioural codes, whereas other schemes combine the two modalities (e.g., the SPAFF) and some focus only on speech (PAICS-R, CCS). For example, “validation” is assessed as affect (non-verbal behaviour) in the RACS, but from speech and non-verbal combined in the SPAFF, or just speech in the CCS. Most codes reflect one person's behaviour to another (e.g., command/direct, criticism, positive physical contact), although nearly all schemes contain concepts such as “validation” which are inherently dependant on the prior behaviour of the other. Only one scheme specifically conceptualises a dyadic behaviour [discourse change; (CECS)].

Developed for adolescents: Only three schemes were described as being developed for adolescents (the RACS, PAICS-R, CECS). The CECS is described as drawing on adolescent-specific theory, the RACS is a combination of the RPC developed for children and the SPAFF developed for adults. The PAICS-R was developed from an adult scheme but describes focusing on behaviours relevant to adolescent-parent conflict. Other schemes were either developed for children and applied to adolescents, or developed to apply to all family relationships (e.g., parent-parent, parent-child, parent-adolescent).

Tasks: Most schemes were developed for problem-solving tasks, specifically conflict resolution tasks where the parent and child identify three areas of disagreement and are asked to discuss them and attempt to reach a resolution. One scheme was developed for a similar “revealed differences” task where parent and adolescent completed a moral judgement task and then discussed the answers where they differed (CECS). The OSLC schemes are described as being suitable for a variety of structured and unstructured settings (e.g., mealtimes). This includes the structured task of planning an activity. The RACS has been used with the Family Assessment Task (69) battery of discussion tasks which includes tasks designed to elicit positive discussion on: encouragement of personal growth (parent and child pick a topic of growth, such as academic achievement, to discuss) and positive recognition of family member (parent and child have a discussion about positive features of the other). No tasks were specifically designed to elicit shared enjoyment.

##### Summary of global coding schemes

Table 3 displays the identified global-coding schemes, with columns summarising whether they were developed for adolescents, their history, what tasks they were developed for, and the type of codes contained. A briefer and less detailed narrative summary of the characteristics of the global schemes is provided below.

**Table 3:** Summary of the characteristics of the identified adolescent-parent global-coding schemes.

Author	Name of scheme	Summary of codes	Made for adoles­cents?	History/theoretical background	Developed for specific population?	Tasks developed for
Allen et al. (24)	Autonomy and relatedness coding system (ARCS)	Global codes: all rated for parent behaviour toward adolescent and adolescent behaviour toward parent: 2 promoting autonomy, 3 inhibiting autonomy, 3 promoting relatedness, 2 inhibiting relatedness. 8-point rating scale (0–4 with.5 increments) with rules based on numbers and types of statements determining rating).	Yes	Developed from Attachment theory [Bowlby (78–80)] applied to adolescents, where the attachment system is activated to provide a sense of “felt security” as opposed to safety [Allen & Land, Allen, Cummings & Davies (81–83)]. In adolescence, the exploratory attachment system is highly activated and the system whereby an individual relies and depends on their attachment figure is reduced (81). The scheme is designed to capture these processes where there is an increase in autonomous behaviour whilst still using the parent as a secure base.	No	Problem solving tasks (conflict discussion and “revealed differences” of moral dilemma task discussion)
Barrett et al. (84)	Macro-coding schedule for parent and child behaviour	Global codes: 7 individual codes (positive & negative, parent & child), 1 parent- only code (positive). 6-point scale.	No	Developed to characterise parent-child interaction in families with a child with obsessive compulsive disorder. Drew on literature suggesting parents may model caution, avoidance or fearfulness [Henin & Kendall (85)], or be strict and overinvolved [Merkel et al. (86)], lack warmth [Ehiobuche, Hoover & Insel (87, 88)], and have high expectations for their children [Hollingsworth et al. (89)].	Yes—clinically referred children	Conflict discussion and discussions around hypothetical ambiguous and therefore anxiety provoking situations the child (e.g., child sees group of children playing a game but they are laughing when child walks over)
Dickstein et al. (90), Hayden et al. (91)	The adapted mealtime family interaction coding system (MICS)	The MICS has 6 dimensions, measuring task accomplishment, communication, affect management, interpersonal involvement, behaviour control and overall family functioning. The dimensions are presented on a 7-point Likert scale ranging from 1 (very unhealthy) to 7 (very healthy).		It is adapted from the McMaster Structured Interview of Family Functioning (McSIFF) and based on the McMaster Model of Family Functioning [Epstein et al. (92)].	Children with chronic illness (but also used with general population)	Observations of family functioning in unstructured, naturalistic situations (specifically meal-time)
Dishion et al. (93)	Coder impressions questionnaire (COIMP)	79 single item description global codes: parental support (parent only, positive), behaviour management (parent only, positive and negative), conflict resolution (parent and child positive and negative) and broader interaction (parent and child, positive and negative). Some specific items assessing antisocial content. 10-point scales	Yes—adolescents and their family	Developed in the Oregan Social Learning Centre (OSLC) based on social learning theory, especially the coercion model of antisocial behaviour [Patterson, Patterson et al. (34, 94)]. Focus on assessing family management and problem solving to understand/predict antisocial behaviour in adolescence.	General population	Designed for a range of discussion tasks [described as the FAST (69)]: planning activity, encouragement of an area of growth for the child (e.g., academic growth), positive recognition of family member, conflict discussion
Feldman (95)	Coding interactive behaviour (CIB)	Codes not specified (manual not openly available). 5-point rating scale.	Yes—multiple versions of the coding scheme, one for adolescents	The coding system measures elements of the theoretical model proposed by Feldman (96). The theoretical model describes how the child and the mother's behaviour influence one another. For instance, how the child and mother's biology, relationships and affective cognition influence one another which then influences overall parenting behaviour	No	Used with a range structured problem solving and discussion tasks, including conflict discussion and positive valence discussions (e.g., plan the best day ever)
Hagstrøm et al. (97)	The tangram emotion coding manual for children (TEC-M)	Global codes: 8 parent codes (positive & negative), 8 child items (positive & negative), 1 dyadic (positive). Frequency score (0–3) and intensity score (1–3) given for each code.	No—developed for children	Designed to assess profiles of emotional regulation in children in the context of parent-child-interactions. Developed based on the theoretical framework of the process model of emotional regulation [Gross (98)] with the five regulatory processes from this model constituting the skeleton of the scoring sheet.	No	Developed for a specific puzzle task designed to evoke emotion regulation behaviours
Hetherington and Clingempeel (99)	Family interaction global coding system (FIGCS)	Global codes: 14 individual codes (parent & child; negative & positive), 2 parental codes (influence and monitoring), 3 child codes (positive & negative). 5-point rating scale. Intensity and frequency rated for each item.	No—families	Scales were based on Baumrind's (1967) parenting typologies (100) and Olson's (Olson et al., 1982) circumplex theory (101).	No	Problem solving tasks
Holmbeck et al. (102)	Family interaction macro-coding system (FIMS)	Global codes: mix of dyadic and individual codes in 3 domains: 15 interactional style, 5 conflict, 8 affect (positive & negative), 3 control (positive & negative), 5 parental behaviours and collaborative problem solving (positive & negative), summary family measures (2). 5-point scale.	No—families of children and adolescents	The scheme is an adaptation of a system developed by Holmbeck et al., Johnson & Holmbeck and Smetana et al. (103–105)). Codes are also based on systems developed by Allen et al. (24, 106), Buhrmester et al. (107)—from work on parenting styles [e.g. Baumrind (100)] Julien et al. (108)—the Interactional Dimensions Coding system, developed to assess conflict and intimacy in martial communication, Levy (109)—maternal overprotection and Paikoff (110)—child scaffolding and problem solving.	No—but first used with samples of children with physical illness	Problem solving and positive event planning tasks
Lindahl and Malik (111)	System for coding interactions and family functioning (SCIFF)	Global codes: 4 family (negative & positive), 1 dyadic (marital communication), 5 parent (positive & negative), 4 child (positive & negative) and 2 categorical family codes. 11 total codes. 5-point scales.	No—child then applied to adolescents	Theoretical foundations for this coding system primarily are family systems [e.g., Boscolo et al. (112)], structural family theory [e.g., Minuchin (113)], and social learning theory [e.g., Patterson (34)]. These theories were used to develop codes that would capture the nature of family interaction patterns and highlight adaptive and maladaptive aspects of family relationships. Developed with children then applied to adolescents, and to triadic as well as dyadic interactions.	General population	Problem-solving tasks
Lyons-Ruth et al. (114)	Goal-corrected partnership adolescent coding system (GPACS)	Global codes: 6 parent (positive & negative), 4 child (positive & negative) and 2 dyadic (positive) codes rated on 5-point scales. 12 total codes. Categorical classification of attachment status made according to rules: 1. Secure, 2. Insecure organised and 3. Disorganised.	Yes	Developed based on Attachment theory specifically applied to adolescents and using observations of parent-adolescent interaction and Adult Attachment Interviews.	No	Reunion and conflict discussion tasks
Melby et al. (115)	Iowa family interaction rating scales (IFIRS)	Global codes: 10 individual characteristic scales (parent & child, positive & negative), 22 dyadic interaction scales (positive & negative), 2 dyadic relational (positive), 15 parenting (positive & negative), 5 individual problem solving (parent & children, positive & negative), 5 group problem solving (positive & negative), 1 group interaction scale. 60 total codes. 9-point scale. Two composites created: collaborative parenting and over-involved parenting.	Yes—adolescents from early adolescence to late adulthood, and their families	Adapted primarily from the FIGCS (Hetherington & Clingempeel, 1992, see section for history) and also draws on social interactional, behavioural (including Patterson's coercive family cycles), or social contextual theories in assessing displays of behaviours and relationship processes at the individual, dyadic, and group levels [Conger, Conger, et al., Conger & Simons, Gottman, Patterson and Patterson et al. (116–119)].	General population	Problem solving tasks and also used in positive discussion tasks
Owen et al. (120)	National institute of child health and human development study of children and youth development (NICHD-SECYD) coding scheme	Global codes: 5 parent (reflecting positive and negative), 4 child (positive and negative) and 1 dyadic (positive) codes.10 total codes. 7-point scale.	No	Developed and extended from the infancy NICHD coding which draws on attachment theory. Extended to include codes related to autonomy promotion [e.g., Ryan et al. (121)], stimulation of cognitive development, and hostility.	No	Problem solving tasks (including conflict discussion and in early adolescence planning/problem solving tasks)
Robin and Foster (70)	Interactive behavior code (IBC)	Global codes: 31 negative communication (dyadic), and 7 positive communication (dyadic). 22/31 negative and 7/7 positive codes are rated absent or present, remainder on a 5-point scale. Modified in Pelham et al. (122) so that every item is rated on a 7-point scale	No—families	Behavioural and family systems theory.	No—but mainly used in clinical populations	Problem solving tasks
Snyder (123)	Macro-level family interaction coding system (MFICS)	Global codes: 3 broad dyadic scales with 55 items: positive engagement (13 items, positive), withdrawal avoidance (17 items, negative), reactivity-coercion (18 items, negative). 5-point scale (1 = not true, did not occur, 5 = clearly evident, very descriptive), designed using an *a priori*, face-valid approach to assess the occurrence of behaviours reflecting positive engagement (20 items) and reactivity-coercion (17 items).	No—children and adolescents	Social learning theory.	No	Problem solving tasks, and a cooperative play activity (a block tower building task)
Vanwoerden (124)	Observational coding system for real-time parent-adolescent mentalising	Global codes: 2 parent codes (positive and negative mentalising), 2 adolescent codes (positive and negative mentalising), and one dyadic (dyadic mentalising).7-point scale.	Yes	Theory on mentalizing and hypo-mentalizing [e.g., Luyten et al., Bateman & Fonagy (125, 126)]	No	Problem-solving (conflict discussion)
Ziv et al. (127)	Conflict task coding system (CTCS)	3 parent codes and 4 adolescent codes on a 7-point Likert scale, assessing conflict tactics.	Yes	The coding system drew on attachment theory, specifically on the work of Kobak et al. (128) and Crowell et al. (129) on attachment in adults and adolescence.	No	Problem-solving (conflict discussion)

Scheme history: schemes drew on a variety of theoretical backgrounds, including social learning theory, psychoanalytic work on ego development and autonomy promotion, attachment theory, family systems theory, structural family systems theory, Baumrind's (100) parenting typologies and Olson's (101) circumplex theory. Some drew on research findings on conflict resolution as well as on parenting of children with anxiety.

Code types: as all schemes are global they combine multiple different units of behaviour (e.g., speech, facial expression, body language), although some schemes rely more on speech (e.g., The autonomy and relatedness coding system (ARCS); (24)) than others which take a more balanced view of all behaviours (e.g., System for coding interactions and family functioning (SCIFF); (111)). There is some variation across schemes in how much the codes reflect an overall impression or “feeling” regarding the interaction, with some comprising specific rules about which point on the scale is assigned [e.g., ARCS; (24)] and others purely relying on global impression (e.g., Coder impressions questionnaire) (93). Some schemes describe all codes as dyadic [e.g., the Adapted Mealtime Family Interaction Coding System; MICS (91)], but most include a mix of ratings about the parent and the adolescent separately, with a smaller number of specific dyadic codes.

Developed for adolescents: Seven schemes were specifically developed for adolescents, or adolescents and their families. These often also drew on adult schemes. The remainder were developed for children and applied to adolescents or developed for both children and adolescents.

Tasks: all schemes were developed for problem solving tasks, mainly conflict discussion but also revealed differences and puzzle tasks. Many schemes were also used with planning tasks, typically planning a family activity. A broader range of discussion tasks which were designed specifically to evoke positive interactions (such as planning “the best day ever”, celebrating a family member) were also used with four of the schemes (the Coder Impressions Questionnaire [COIMP, (93)], the Coding Interactive Behavior [CIB, (95)], the Family Interaction Macro-coding System [FIMS, (102)], the Iowa Family Interaction Rating Scales [IFIRS, (115)].

##### Overall summary

Less micro- compared to global coding schemes were identified, and many of the identified micro-schemes were further developments or extensions of existing schemes. Very few of the existing micro-coding schemes were developed specifically for adolescents and so there is less influence of adolescent specific theory on their development. The majority of micro-schemes were mostly based on social learning theory principles and were largely designed for and employed with problem solving or conflict discussion tasks. Global coding schemes were more commonly developed specifically for adolescents and drew on a wider range of theory. Whilst conflict and problem-solving tasks still predominate, these schemes have more often been employed with tasks designed to evoke positive interaction. This underscores the importance of drawing on both global and micro-schemes in the development of a new comprehensive and relevant adolescent coding scheme.

#### Scoping public engagement

The mobile event visited 5 unique locations in the South-West of the UK, in busy family areas during the school holidays, including a beach front, local parks in residential areas out of the city, central areas directly in the city centre (close to fairgrounds and museums) and rural “car boot” sales. The areas varied in deprivation indices and the ethnicity of the populations. The mobile research event received active responses (*in the form of a written response on a white board and voting for proposed measures using tokens and slot boxes*) from 234 young people and/or parents. Where votes were collected, we recorded whether the vote was from an adolescent, a young adult (18–25) or a parent.

Acceptability of wearable head cameras: We asked families about the use of wearable head cameras as well as several other wearable technologies including rings, watches, actigraphy and on-body cameras (raw data in Supplementary file S2). Families had mixed views about taking part in research using wearable cameras: some were initially sceptical, but once it was explained that the proposal would be to use them for short-term interactions, not all-day surveillance, the method was deemed acceptable. Young people especially favoured seeing the footage capture from their own camera which represented the world through their eyes.

**Figure 3:**
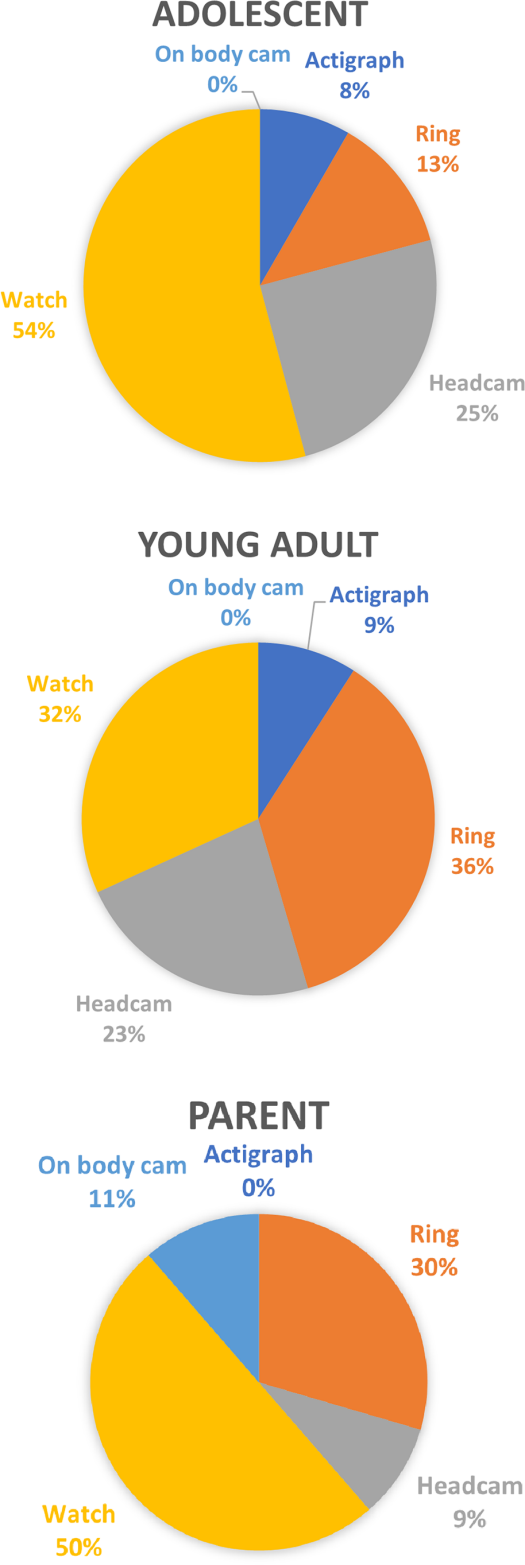
Summary of adolescent, young adult and parent views on acceptability of wearable technology.

We also heard that the advent of body worn cameras used by police and in security settings has led to a sense of being watched in young people. At the heart of people's concerns was not being recorded *per se*, but the *imbalance;* in the police/security context, cameras are *only* worn by those in authority, and these cameras capture one perspective only. By contrast, in our proposed measure, both perspectives are represented. Young people were positive about collecting footage that showed the events through their eyes, not just footage watching them from another person's perspective. As shown in Figure 3, when asked about the acceptability of different wearable technologies in research, 25% of adolescents were happy to wear a wearable camera. Given that the other wearable measures were far less intrusive and mainstream (watches and rings), this demonstrates a reasonable engagement with wearable cameras. For purposes of illustration, Figure 1 shows two adolescents playing a card game wearing a wearable camera.

**Figure 4:**
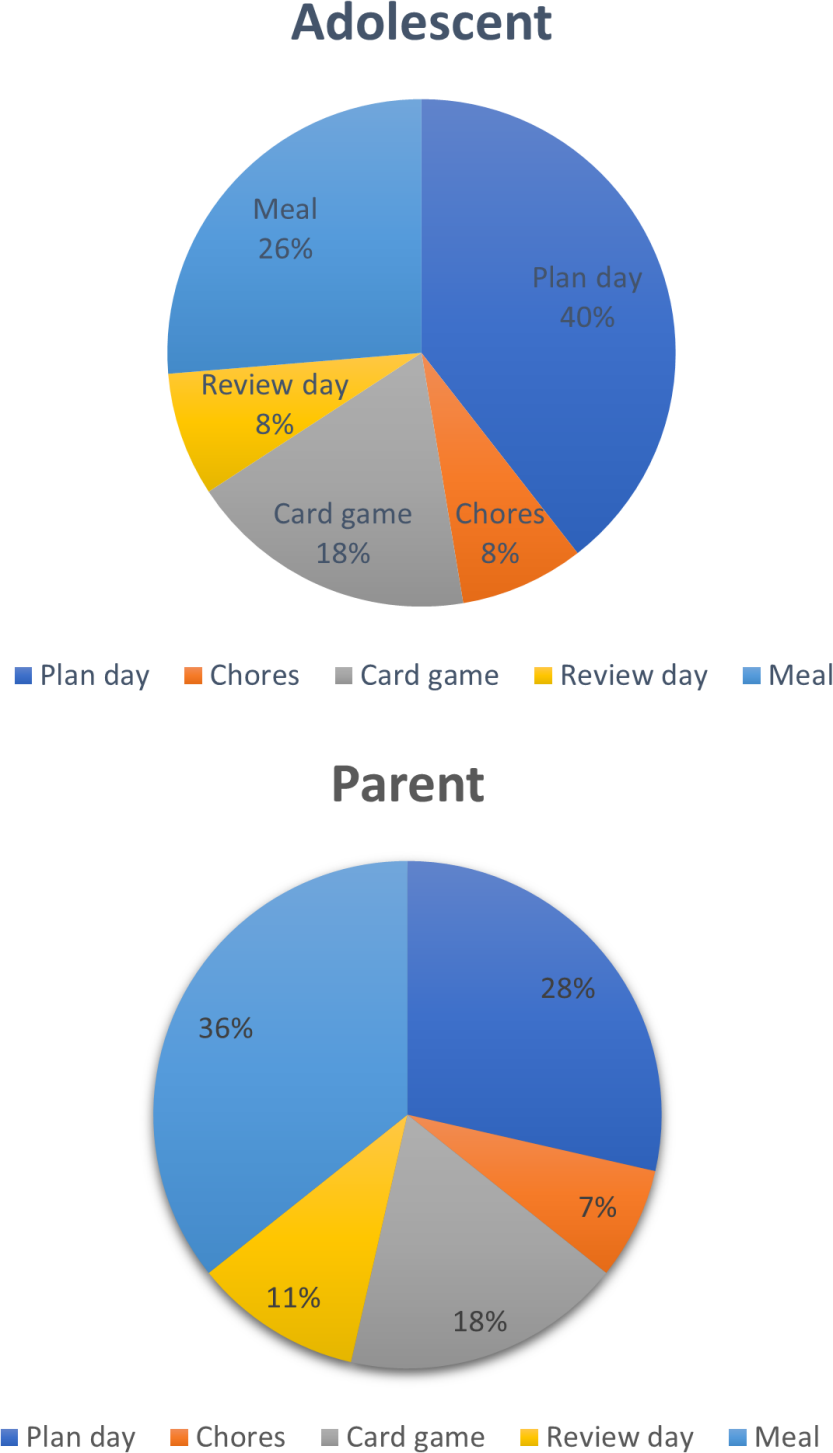
Summary of adolescent and parent report views on task to capture adolescent-parent interaction at home.

Task to adequately capture interactions at home: As shown in Figure 4 (raw data in Supplementary file S2), the most voted for task to adequately capture interactions at home was a discussion task to plan the day or week, with 40% of adolescents choosing this task, followed by 26% choosing meal-time, 18% playing a card or board game, 8% completing chores together and 8% reviewing their day. Parents voted for mealtimes, followed by planning their day or week and then playing a game. Many families described that playing a board or card game was not something that they would typically do, but that they would be happy to do it. We brought an example game called “Sussed” (130), a conversation-starter card game specifically developed for modern family interactions (further described below in the pilot section). Many parents and adolescents enjoyed trying out the task.

**Figure 5:**
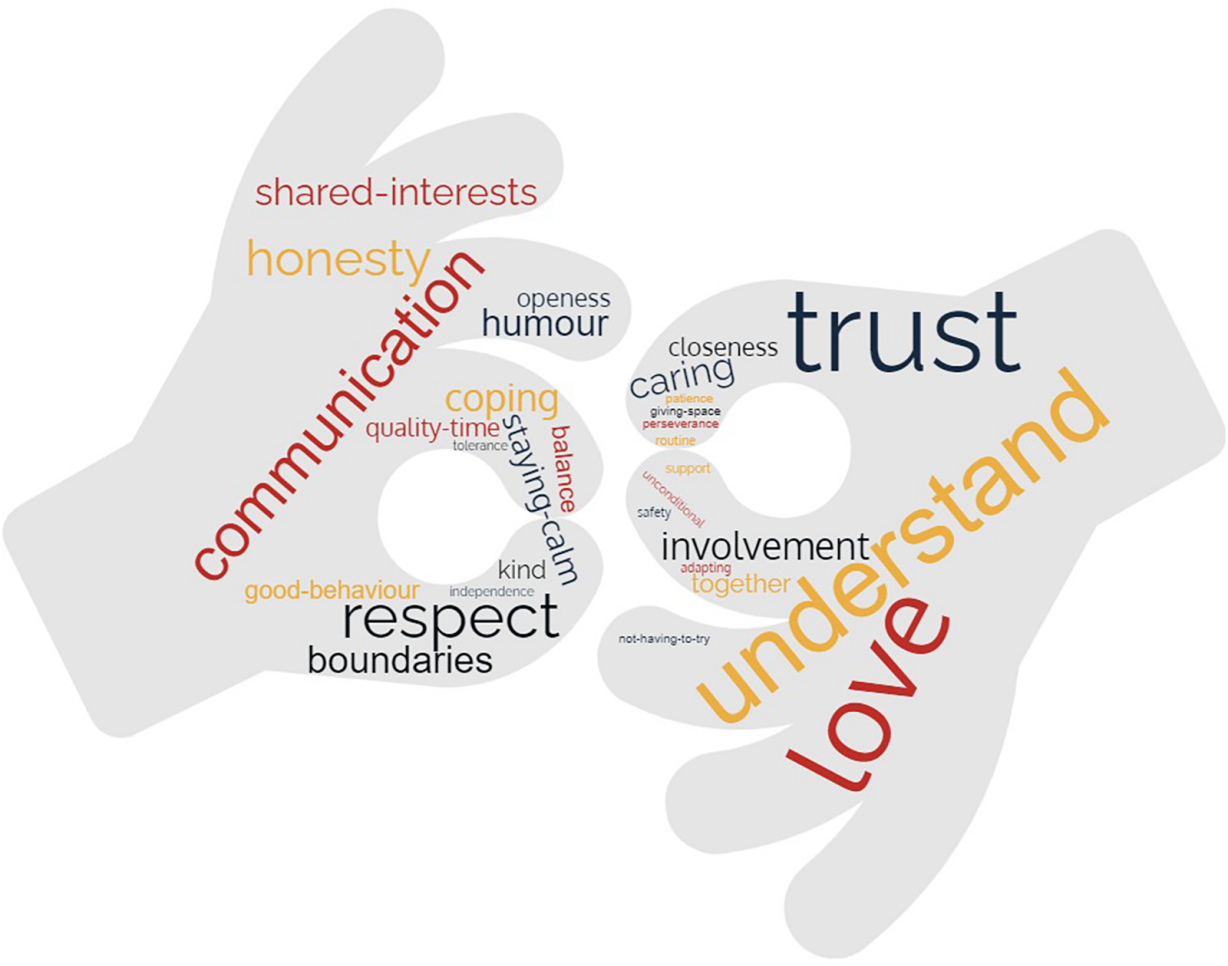
Word cloud presenting the relationship terms reported by adolescents and parents.

Constructs: Figure 5 presents the terms provided by parents and young people in a word cloud (raw data is presented in Supplementary file S3). “Understanding” and “trust” were described most often, followed by love, caring, communication and respect.

## Part 2—Protocol for further work

Below we set out the next steps for the development of observational capture and coding of adolescent-parent interactions.

### Systematic review of measurement properties of coding schemes

A systematic review of the measurement properties of existing observational measures of adolescent-parent measures is required, to better understand the strengths and weaknesses of existing measures, as well as to identify gaps. We have registered the review with PROSPERO (CRD42023397423) and our protocol is publicly available at [https://www.crd.york.ac.uk/prospero/display_record.php?ID = CRD42023397423]. In brief, we will conduct a new search, informed by the work presented earlier. We will search MEDLINE, EMBASE, APA PsycINFO, Web of Science (Core Collection), ProQuest Dissertations and Theses Global, Scopus and CINAHL, limited to English language, without date limits. In addition, we will search for unpublished studies on Google Scholar and PsyArXiv. The reference lists of eligible studies will be manually screened to identify other relevant studies.

We will search for studies investigating psychometric properties of observational measures of adolescent-parent interaction. We define such measures as any instrument in which a coder evaluates any aspect of adolescent-parent interaction on the basis of observed behaviour, utilising some form of a coding system, which can be micro or global. We will include: any empirical study reporting psychometric properties of an observer-rated measure of adolescent-parent interaction; the mean age of children in the sample must be between 10 and 19; by parent, we refer to biological and adoptive parents, as well as carers whose role is that of a primary caregiver to the child (e.g., foster carers); studies must be in the English language; we will include both published and unpublished studies that are available as completed full-text reports, including dissertations, book chapters or pre-print papers. We will exclude studies of interactions with grandparents or any non-primary caregiving role, as well as commentaries, letters, conference abstracts and review.

The aim of the review is to identify the best measures of adolescent-parent interactions based on their psychometric properties, using the COnsensus-based Standards for the selection of health Measurement INstruments (COSMIN) (131). We will utilise the COSMIN tools for appraising study quality, adequacy of measurement properties and overall strength of evidence, which are freely available at www.cosmin.nl. We will assess content validity, structural validity, internal consistency, cross-cultural validity, reliability, measurement error, criterion validity, and responsiveness. By identifying the existing measures with the best evidence of adequate psychometric properties, we will be able to give greater weight to such measures in terms of informing our own task and coding scheme.

### Construct definition and measure development through focused public engagement workshops

We will conduct a series of workshops with adolescents, parents and health professionals, including an interactive live performance with creative arts organisation Made by Mortals (https://www.madebymortals.org/) to operationalise and “bring to life” the constructs identified in the scoping public engagement. The adolescent workshop will include some adolescents with lived experience of mental health problems and accessing mental health services as well as parents and professionals.

In contrast to traditional approaches to consultation through focus groups, we believe that the use of an arts-based approach will provide a more engaging and less abstract way to elicit views. A key strength is that the creative arts materials presented will be based on the story of a family who will represent numerous lived experiences, without disclosing any one story personally. Attendees at these workshops will be invited to share ideas relating to these stories, which connect to themes arising in our earlier public engagement work.

The workshops will include the use of pre-recorded audio and video clips, as well as use of drama performance and opportunities to experience the task and wearable camera technology. We will conduct workshops at various stages in the development of the interaction measure, with the following aims:

1. To inform the development of the coding system, particularly through exploring the extent to which theoretical constructs derived from the literature align with the “experience-near” perspectives of adolescents and parents.
2. To inform the choice of task for the measure.

Harmonisation of information from existing coding schemes with information from public engagement with adolescents and parents

This will follow a stepped process:

1) Extract all codes from the identified observational parent-interaction coding schemes, including both higher-order (e.g., autonomy promotion) and lower-order (e.g., positive affect) concepts. Codes from global coding schemes will be assessed for suitability of use as micro-codes or whether they require separation into multiple concepts.
2) Research team will perform initial clustering of common and related codes and concepts to identify a taxonomy of constructs. This will involve additional broad literature searching (including fields outside psychology such as philosophy and economics) on key constructs to support operationalisation.
3) Consultation with an “expert panel” of clinicians, academics and a PPI panel of adolescents and parents to appraise the content validity of the clusters and resolve any queries or lack of consensus on concepts from the previous step.

### Initial pilot of the wearable camera methodology to provide proof of concept and initial validation study

#### Methods of data capture through use of wearable cameras

We propose that recent techniques for measuring parent-infant interaction from a first-person perspective using wearable cameras within the family home setting (47, 26) could be adapted to investigate adolescent-parent interaction. The advantages of such an approach are:

• Increased ecological validity if used in the home setting, without the need for a researcher to be present during the interaction.
• Content Validity
  ∘ The face-to-face view of the wearable cams captures full face view of both interactors better than mounted cameras in one position only (see Supplementary Figure S1). This allows more subtle detections of facial expressions and vocalisations.
  ∘ The unique perspective allowing researchers to capture interaction “through the eyes” of the young person and parent (26). Pearson and colleagues have previously established that mother-infant interaction can be reliably observed at home using this methodology (26, 132).
  ∘ Potential use of face-reader technology to automate some aspects of coding, thereby dramatically reducing coding time.
• Therapeutic potential
  ∘ This approach has significant potential for future development into video-feedback intervention.
  ∘ For example, the approach is currently being used to feedback to new mums in South Africa (see Cantrell et al. in this edition). Of note a goal of parents is often whether their child looks at them/knows them, the side-by-side view of mum’s perspective and child perspective allows more explicit observation of key moments that can be “re-lived”, thus reinforcement of key connecting behaviours.

*Pilot study aims:* (1) Assess feasibility and acceptability of the wearable camera methodology in a general population sample (2) Collect footage to apply micro-coding scheme to.

### Sample

The Wirral Child Health & Development study (WCHADS) is a prospective birth cohort of mothers, fathers and their first-born children based in the Wirral, UK [see Sharp et al. (133) and https://www.liverpool.ac.uk/population-health/research/groups/first-steps/ for further details]. The study has completed 13 waves of data collection since birth, including observations of parenting in the lab or at home with a researcher present at ages 6 months, 14 months, 2.5 years 3.5 years, 7 and 9 years. At age 11–12 (*N* = 743) and age 12–13 (*N* = 724) families completed postal questionnaires only. The sample are currently aged 13–15 years.

### Procedure

The whole sample will be approached via email to invite a volunteer sample of 50 families to participate in the pilot. The first 50 families who express interest will be recruited. Families will be visited by a researcher to drop off the cameras and explain the procedures, and an appointment arranged for the researcher to return to collect the cameras. Primary caregivers and adolescents will complete two tasks in their own time at home, and complete questionnaires on the acceptability of the wearable cameras and the tasks and validated questionnaires assessing relationship quality.

### Measures

#### Observations of adolescent-parent interaction.

##### The head-cameras

The head-cameras (shown in Figure 1) are small circular cameras attached to a mount on an elastic adjustable headband. The camera devices are off the shelf body cameras. The headband mounts were created by an experienced user centred design company (Kinneir Dufort, KD) to ensure suitable positioning of the camera. Footage is recorded on to a micro SD card.

##### The tasks

Parents and adolescents will be asked to record themselves completing two tasks at home. The choice of task for this initial pilot was informed by the scoping PPI work, review of the literature, and consideration of the potential issues with using conflict discussion tasks at home and to evoke positive interaction. In the first task, parents and adolescents are asked to play the commercially available card game “Sussed” which was developed by researchers and funded by the UKRI (130). “Sussed” is a conversation starter game designed to improve social health by players answering multiple choice questions about each other, allowing players to find out how others see them, talk about how others see them and understand why people think or feel a certain way. Example question: Who do I most like to work with? (A) People who are the same as me, (B) People who are different to me, (C) I prefer to work on my own. Parents and adolescents will be provided with 10 cards which were identified from the standard yellow Sussed set (suitable for age 6+) and the green emotional health Sussed set (suitable for age 8+) by A level and first year undergraduate students. Parents and adolescents will be asked to play the game and record for 10 min. In the second task, the parent and adolescent are asked to share a drink or a snack and discuss their plans for their week, and record for 5 min.

##### Questionnaires

Self-developed questionnaires to assess acceptability of the methodology: Questions ask about the acceptability of using the wearable cameras, the tasks they were asked to complete, and their thoughts on collecting footage from the first-person perspective of both mother and adolescents. Parent and adolescent report.

Adolescent-parent relationship quality: the Network of Relationships (NRI) “seeks safe haven” subscale (support seeking from parent, parent and adolescent report) (134), the brief version of the Parental Feelings Questionnaire (positive and negative feelings about child, parent report and adapted for adolescent) (135) and the trust subscale of the Inventory of Parent and Peer Attachment (IPPA, adolescent report) (136).

### Planned output

Feasibility indices: this will include proportion of families invited to use the wearable cameras that agree to take part, of those who agree how many provide footage, we will also record reasons for not managing to obtain recorded footage, challenges in both the initial recruitment and the recording of interactions. For example, a similar protocol was developed for parents and infants in the ALSPAC study (Using a wearable camera at home—Children of the Children of the 90s—YouTube). Such information can be used to inform protocols for use in cohort studies, time needed for researcher time and likely uptake. Potential cohorts include ALSPAC-G2 (137).

Proof of concept: recorded footage will be used to develop the adolescent-parent coding scheme by testing application of identified “behaviour groupings”/parent-child interaction codes to the videos in order to refine definitions.

1. Initial reliability and validation study: inter-rater reliability of the newly identified coding scheme will be assessed. Self-reported relationship quality will be used to provide an initial exploration of construct validity of the observed adolescent-parent relationship constructs. Given the sample size of 40 power will be too low to provide strong statistical evidence for associations between self-report and observations, which are expected to be around effect sizes of 0.3 based on previous studies linking observed and self-report of parenting (25).

### Co-design of a comprehensive and relevant coding scheme in consultation with an expert panel and lay/PPI panel

Agreed behavioural domains will be operationally defined and incorporated into an Observer XT coding structure. We will apply the coding structure to the WCHADS videos and consult with experts and young people as “coders” to determine face validity regarding whether the defined behaviours can be applied to live behaviour examples. The aim is that multiple, diverse coders can use the coding scheme to accurately capture all behaviours and dyadic behaviours observed in real life interactions, and that multiple coders give the same code for the same observation (inter-rater reliability). The coding scheme will be refined during this process and may include updated codes to capture behaviours that emerge and redefinition of behaviours that do not reach inter-rater-reliability. Once finalised we will publish an open access, co-developed, fully illustrated manual of behavioural codes and observer syntax in the same way that we have for micro-coding in the early years (47).

## Conclusion

The initial work presented in this paper suggests that there is a need to co-design both new methods of data capture, and new coding schemes, to assess adolescent-parent interaction. In our scoping review, we identified that numerous observational measures have been developed over the years, but fewer coding schemes were developed specifically with adolescents in mind this was particularly the case in existing micro-coding schemes. None of the coding schemes were co-designed with adolescents. This is important since adolescence constitutes a unique period in which parent-child relationships are in transition in a variety of ways, such as becoming more symmetrical as adolescents develop more independence and autonomy (4), therefore requiring adolescent-specific theory to guide their development.

Our initial review of the evidence indicates that negative and conflictual parent-child interaction processes are well characterised, largely by the application of social learning theory principles such as Patterson's coercive cycle (34). In the programme of work described here we place emphasis on characterising the more positive aspects of adolescent-parent interaction. Positive adolescent-parent relationships are associated with greater adolescent well-being (38), and the most promising relational interventions are strengths based (138, 139). Consistent with a focus on problematic interactions, we note that a large number of data capture methods utilise problem-solving or conflict-related tasks. Whilst conflict tasks are used to code positive adolescent-parent interaction qualities, they are less likely to evoke positive interaction qualities (14, 15), and the negative affect and arousal which accompanies conflict (29–31) may mean they are not best suited to assess positive interactions. In addition, measures eliciting conflict may be less suitable for use in the home context, in the absence of researchers.

Planning activities were also quite commonly used, particularly in studies employing global scales. Planning discussions involves some problem solving and negotiation but are not conflict based. We also identified some structured discussion tasks designed to elicit positive interactions, including celebrating a family member and parent encouragement of an area of personal growth in the child or planning a fun family activity (69). In our public engagement work we asked families what the best contexts at home would be to observe their most natural adolescent-parent interaction at home. Planning their day or week was the most voted for task. Given the focus in infancy observation on tasks which elicit shared enjoyment, and our aim to observe positive adolescent-parent interaction, we also suggested a card game task which most families found acceptable. There has been very little consideration in the literature on the content and ecological validity of different observational tasks, particularly in relation to characterising positive interactions, in adolescence. This is essential for developing a method to adequately characterise adolescent-parent relationships.

In our public engagement work, we found that parents and adolescents were open to the possibility of utilising technology such as wearable cameras or heart-rate monitors and the possibility of automated technology coding behaviours. In terms of content validity, we have found that the most relevant concepts expressed in PPI work such as Trust and Understanding are not explicitly covered in existing coding or assessment tools. These higher-order concepts are likely represented by multiple individual codes contained in existing schemes. Careful conceptual and practical mapping of top down and bottom-up content will be required to ensure new coding schemes capture what is seen as most relevant.

Our work thus far has some limitations. Firstly, since the literature on observational adolescent-parent measures appears to use a wide range of terms, it is probable that our scoping review may have missed some existing measures. In addition, as most observational coding schemes are not published and can be difficult to locate, our review is likely not complete. Secondly, whilst our public engagement work will have reached individuals not usually approached by researchers and contained a diversity of class and ethnicity, it was exclusively conducted in the South-West of England. Future public engagement work should be conducted in other regions of the United Kingdom prior to developing the coding scheme or undertaking formal validation studies. Furthermore, we are currently evaluating the feasibility and acceptability of the measure in a community sample of adolescents, which will shed light on the potential use of the measure for research purposes in cohort studies. As discussed earlier in this paper, the use of the measure for different purposes and different populations requires additional evidence to support each specific use. In particular, the use of the measure in clinical samples, either for research or therapeutic purposes, will require separate evaluation in research studies. The suitability of the measure for individuals with learning disabilities or an autism spectrum condition also requires evaluation.

In summary, the work presented and planned here represents the first attempt to truly co-create a method to assess parenting in adolescence, combining bottom-up processes to harness insights from adolescents and parents, with top-down approaches to utilise and build on existing theory and research. By using creative approaches to public engagement, as well as rigorous plans for psychometric investigation, we hope to develop an observational measure that captures salient aspects of adolescent-parent interaction using novel technological methods, which can be used across a range of research and therapeutic settings.

## Data Availability

The original contributions presented in the study are included in the article/**Supplementary Material**, further inquiries can be directed to the corresponding author.
