# Supplementary material for: Through each other's eyes: initial results and protocol for the co-design of an observational measure of adolescent-parent interaction using first-person perspective
Source: Front Child Adolesc Psychiatry. 2024 Mar 4;2:1214890. doi: 10.3389/frcha.2023.1214890 (PMC11748891; doi:10.3389/frcha.2023.1214890)

Supplementary Figure 1: Image displaying the first-person view from a mother and adolescent wearing the head-cameras


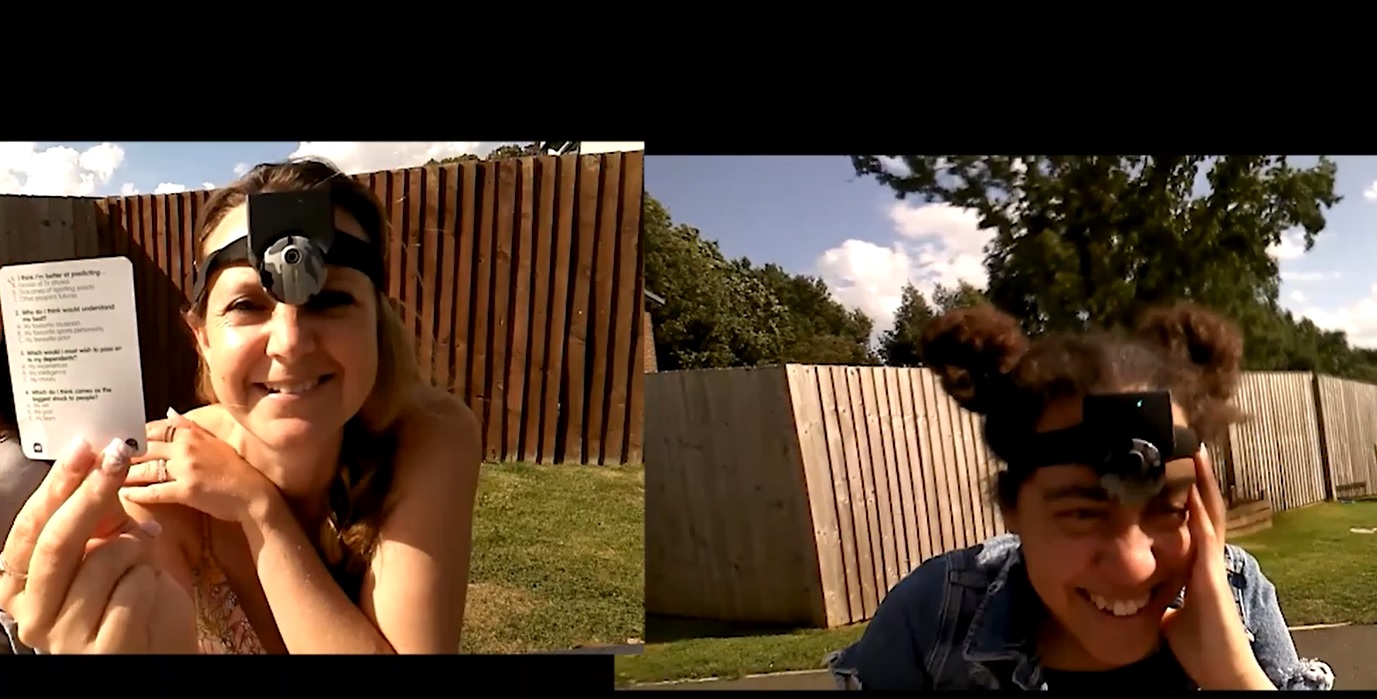

Supplement: Supplementary file 1 [file Datasheet1.zip › Supplementary Figure 1.DOCX]
