# Supplementary material for: Through each other's eyes: initial results and protocol for the co-design of an observational measure of adolescent-parent interaction using first-person perspective
Source: Front Child Adolesc Psychiatry. 2024 Mar 4;2:1214890. doi: 10.3389/frcha.2023.1214890 (PMC11748891; doi:10.3389/frcha.2023.1214890)

**Supplementary materials 1**

All search terms used for the scoping review of existing schemes

Search: **parent adolescent observed interaction coding behaviour**

("parent s"[All Fields] OR "parentally"[All Fields] OR "parentals"[All Fields] OR "parented"[All Fields] OR "parenting"[MeSH Terms] OR "parenting"[All Fields] OR "parents"[MeSH Terms] OR "parents"[All Fields] OR "parent"[All Fields] OR "parental"[All Fields]) AND ("adolescences"[All Fields] OR "adolescency"[All Fields] OR "adolescent"[MeSH Terms] OR "adolescent"[All Fields] OR "adolescence"[All Fields] OR "adolescents"[All Fields] OR "adolescent s"[All Fields]) AND ("observability"[All Fields] OR "observable"[All Fields] OR "observables"[All Fields] OR "observation"[MeSH Terms] OR "observation"[All Fields] OR "observe"[All Fields] OR "observed"[All Fields] OR "observer"[All Fields] OR "observer s"[All Fields] OR "observers"[All Fields] OR "observes"[All Fields] OR "observing"[All Fields] OR "watchful waiting"[MeSH Terms] OR ("watchful"[All Fields] AND "waiting"[All Fields]) OR "watchful waiting"[All Fields] OR "observations"[All Fields]) AND ("interact"[All Fields] OR "interactant"[All Fields] OR "interactants"[All Fields] OR "interacted"[All Fields] OR "interacting"[All Fields] OR "interaction"[All Fields] OR "interactional"[All Fields] OR "interactions"[All Fields] OR "interactive"[All Fields] OR "interactively"[All Fields] OR "interactives"[All Fields] OR "interactivities"[All Fields] OR "interactivity"[All Fields] OR "interacts"[All Fields]) AND ("clinical coding"[MeSH Terms] OR ("clinical"[All Fields] AND "coding"[All Fields]) OR "clinical coding"[All Fields] OR "coding"[All Fields] OR "coded"[All Fields] OR "codes"[All Fields] OR "codings"[All Fields]) AND ("behavior"[MeSH Terms] OR "behavior"[All Fields] OR "behavioral"[All Fields] OR "behavioural"[All Fields] OR "behavior s"[All Fields] OR "behaviorally"[All Fields] OR "behaviour"[All Fields] OR "behaviourally"[All Fields] OR "behaviours"[All Fields] OR "behaviors"[All Fields] OR "pattern"[All Fields] OR "pattern s"[All Fields] OR "patternability"[All Fields] OR "patternable"[All Fields] OR "patterned"[All Fields] OR "patterning"[All Fields] OR "patternings"[All Fields] OR "patterns"[All Fields]

Supplementary Figure 1: Image displaying the first-person view from a mother and adolescent wearing the head-cameras


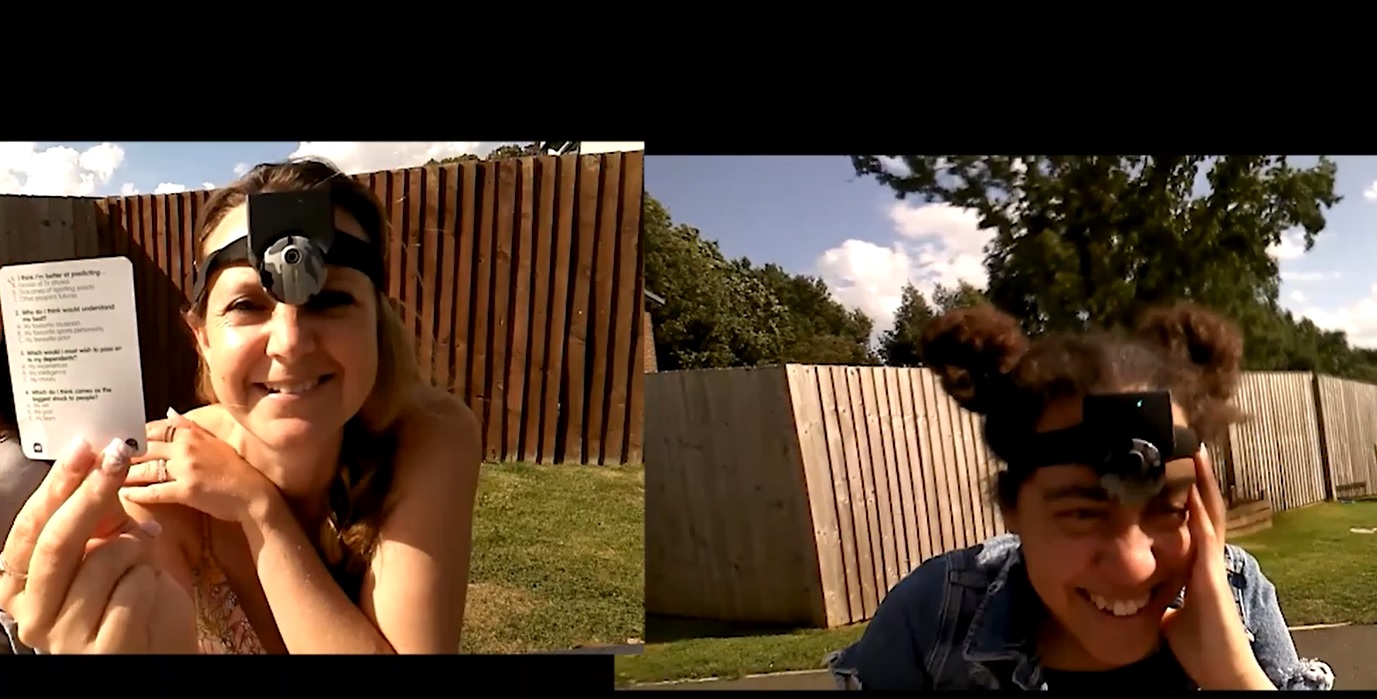

Supplement: Supplementary file 1 [file Datasheet1.zip › Supplementary File 1.DOCX]
